# Auditory Perceptual Exercises in Adults Adapting to the Use of Hearing Aids

**DOI:** 10.3389/fpsyg.2022.832100

**Published:** 2022-05-18

**Authors:** Hanin Karah, Hanin Karawani

**Affiliations:** Department of Communication Sciences and Disorders, Faculty of Social Welfare and Health Sciences, University of Haifa, Haifa, Israel

**Keywords:** hearing aids, age-related hearing loss (ARHL), speech in noise, aging, speech perception, hearing impairment

## Abstract

Older adults with age-related hearing loss often use hearing aids (HAs) to compensate. However, certain challenges in speech perception, especially in noise still exist, despite today’s HA technology. The current study presents an evaluation of a home-based auditory exercises program that can be used during the adaptation process for HA use. The home-based program was developed at a time when telemedicine became prominent in part due to the COVID-19 pandemic. The study included 53 older adults with age-related symmetrical sensorineural hearing loss. They were divided into three groups depending on their experience using HAs. Group 1: Experienced users (participants who used bilateral HAs for at least 2 years). Group 2: New users (participants who were fitted with bilateral HAs for the first time). Group 3: Non-users. These three groups underwent auditory exercises for 3 weeks. The auditory tasks included auditory detection, auditory discrimination, and auditory identification, as well as comprehension with basic (syllables) and more complex (sentences) stimuli, presented in quiet and in noisy listening conditions. All participants completed self-assessment questionnaires before and after the auditory exercises program and underwent a cognitive test at the end. Self-assessed improvements in hearing ability were observed across the HA users groups, with significant changes described by new users. Overall, speech perception in noise was poorer than in quiet. Speech perception accuracy was poorer in the non-users group compared to the users in all tasks. In sessions where stimuli were presented in quiet, similar performance was observed among new and experienced uses. New users performed significantly better than non-users in all speech in noise tasks; however, compared to the experienced users, performance differences depended on task difficulty. The findings indicate that HA users, even new users, had better perceptual performance than their peers who did not receive hearing aids.

## Introduction

Individuals with sensorineural hearing loss generally use hearing aids (HAs) to compensate for hearing loss. Despite the advanced technology of HAs, many individuals demonstrate “hearing aids in the drawer” phenomena (Bisgaard and Ruf, 2017), where they often remove one HA or become dissatisfied with the assistance they receive from it (Hoppe and Hesse, 2017; Johnson et al., 2018). The most common reason people do not use their HAs is that they did not provide an additional benefit, particularly in noisy environments (McCormack and Fortnum, 2013). Since the adaptation period to HAs is very important, the current study evaluated a program that offers home-based auditory exercises in noise to assist with the adaptation process of HAs and examined the benefits of the use of such exercises in the subjective hearing ability reported by the listeners. In addition, the current study examined perceptual differences between adults with non-rehabilitated hearing loss and those who used hearing aids to compensate for their loss.

Subjective benefits of HAs are inconsistent, especially when assessed for noisy listening conditions. Previous studies showed that subjective benefits of HAs are low, especially in noisy environments (Kochkin, 2000; Hartley et al., 2010). Karawani et al. (2018a) showed that satisfaction with HAs increased over the course of 6 months. Verma et al. (2017) measured subjective benefits of the use of HAs at 6 months, 1 year, 1.5 years, and 2 years using the Hearing Aid Benefit Questionnaire, and showed high satisfaction scores in aided situations. However, benefits varied depending on whether the listening background was quiet, noisy or music. The most difficult environments and the lowest scores were noted when speech was presented in a background noise condition compared to quiet or music conditions. Therefore, although HAs have been shown to improve cognitive and cortical function (Karawani et al., 2018b; Glick and Sharma, 2020; Karawani et al., 2022), and perceptual abilities (Munro and Lutman, 2003; Lavie et al., 2015; Megha and Maruthy, 2019; Wright and Gagné, 2020), the benefits of speech in noise processing are limited (Sweetow and Sabes, 2006; Humes et al., 2009, 2013; Kaplan-Neeman et al., 2012; Karawani et al., 2018a; Bieber and Gordon-Salant, 2021).

A recent systematic review suggested that “individuals with hearing impairment seem to benefit the most using a combination of sensory rehabilitation with HAs and auditory training to enhance auditory rehabilitation” (Stropahl et al., 2020, page 1). Given that many HA users spend years listening to attenuated speech (caused by the degradation of their own hearing) before obtaining amplification, it is possible that a newly fitted (new) HA user might benefit from an additional auditory perceptual program focused on learning how to interpret newly amplified speech (Burk et al., 2006).

Studies with new HA users reported auditory training benefits. For example, Yu et al. (2017) recruited two new HA users and worked with them on auditory cognitive training ReadMyQuips (RMQ) software for 8 weeks. The stimuli were syllables/ba//ga//la/, which were presented in different conditions (auditory only, visual only, auditory and visual congruent, and auditory and visual incongruent). The results showed improvements in the connectivity of brain regions after auditory training. A study by Rao et al. (2017), also used the RMQ for a 4-week training course and showed that first-time (new) HA users with mild-to-moderate hearing loss showed improvements in speech perception in noise and improvement in auditory selective attention. A case study by Jo et al. (2013) examined a 70-year-old man who had used HAs for 5 years. He was enrolled in an 8-week auditory training study. The Amplification in Daily Life and International Outcome Inventory for Hearing Aids Questionnaires were used (Cox and Alexander, 1999, 2002). Environmental sounds, consonants, sentences, and a crossword quiz were used as the key training stimuli. The recognition abilities of the four conditions were compared before and after training. The results showed that the training benefit was most apparent in a noisy environment compared to a quiet condition. Further, HA satisfaction was also greater after the auditory training course. Lavie et al. (2013) evaluated one-on-one dichotic listening sessions with new HA users and found that new adult HA users who received one-on-one live speech sessions during the adaptation period showed significant improvements in dichotic listening scores compared to the group without the exercises.

Although these studies demonstrate a positive effect of training, the literature also shows that auditory training does not always induce large effects, especially for generalization to untrained sounds (for a review, see Lawrence et al., 2018). Karawani et al. (2016) developed a home-based training program that was administered for 4 weeks on non-HA users with mild-to-moderate age-related hearing loss, and reported improvements in speech in noise, time compressed and competing speaker conditions. However, generalization to untrained stimuli and tasks was less effective. In addition, improvements from training can be specific to trained stimuli, and may or may not depend on the length of the training course, and on other factors (see reviews by Lawrence et al., 2018; Bieber and Gordon-Salant, 2021). Tye-Murray et al. (2017) examined the benefits of auditory training in 47 experienced HA users, who were divided into two training groups, that differed in the time course of training. One group received training in the lab twice a week for 10 weeks, and the other group received more intensive training where they had to train 5 days a week for 2 weeks in the lab. Speech perception tests (word identification, word discrimination, and sentence recognition) were administered before, immediately after, and 3 months after training. The results revealed that speech perception improved in both groups with no effect of the duration (2 weeks vs. 10 weeks) or the extensiveness (5 days vs. 2 days a week) of the training protocol.

With improvements in technology, computer-based auditory training programs have been implemented both experimentally and clinically. Researchers have examined the effects of computerized auditory training programs for adults using HAs (Burk et al., 2006; Sweetow and Sabes, 2006, 2007; Miller et al., 2008). Sweetow and Sabes (2006) used a home-based computer training program – Listening and Communication Enhancement (LACE) with experienced HA users (>6 months) and reported that most improvements occurred during the first 2 weeks of training. In another study by Stecker et al. (2006), new and experienced HA users (10–21 months) participated in a home-based computer training of syllable identification in noise for 8 weeks, with five sessions each week. Similar improvements were observed in new and experienced users. Moreover, a study by Olson et al. (2013) with 29 new (<6 months of HA use) and experienced (>2 years of HA use) adult HA users and a group of non-users evaluated auditory training, using the LACE DVD program for 4 weeks. The program included speech in noise, rapid speech, and competing sentences tasks. The results revealed that both HA user groups improved in the three tasks, and the largest effect was observed in the new HA users. A later study by Saunders et al. (2016) examined the use of the LACE as a supplement to the new HA adaptation period and compared new users (<6 months) with experienced users (>1 year). The study groups were divided into 4 intervention groups: LACE training using a 10-session DVD format for 2 weeks, LACE training using a 20-session computer-based format for 4 weeks, a placebo auditory training group consisting of 10 h of active listening to digitized books on a computer for 4 weeks, and an educational counseling control. Participants were tested on perceptual and cognitive measures and completed self-assessment questionnaires [the Abbreviated Profile of Hearing Aid Benefit (APHAB) and the Hearing Handicap Inventory (HHI)]. The outcomes were tested immediately after the training and 6 months later. The authors [unlike previous reports by Sweetow and Sabes (2006) and Olson et al. (2013)] reported non-significant differences across groups and across interventions, concluding that particular use of the LACE listening program did not induce benefits. Taken together, pre- and post-outcome measures used to test learning, as has been shown in the previous studies mentioned above, may vary across protocols, and may account for the variability in the results. The current study aimed to compare between groups by investigating their performance along more comprehensive auditory tasks, and not only in pre-post-outcome measures.

Although these studies were informative, with some positive effects from training, the literature also shows that auditory training may have minimal-to-no effects, especially for generalization to untrained sounds (Saunders et al., 2016; Lawrence et al., 2018). Previous studies have shown that one-on-one coaching and clinic/lab-based interventions in the adaptation period are beneficial for HA use (Lavie et al., 2013). Others have suggested that educational programs on the benefits of HAs could supplement the clinical rehabilitation process (Ferguson et al., 2016, 2021) and may show benefits in the adaptation period of HA use (Maidment et al., 2020).

### The Current Study

Because we are in the era of telemedicine (Tao et al., 2021) and due to adaptations required due to the COVID-19 pandemic, we developed an online, home-based auditory program designed to assist individuals in the adaptation process of HAs, in particular during the first 6 months of adaptation to the amplification aid. The goal of this study was to examine the effectiveness of perceptual auditory exercises for HA users comparing experienced users with new users and non-hearing aid users in self-report measures of hearing ability, and compare their performance on the different tasks in quiet and under background noise.

As has been seen in previous studies, the period needed to observe improvements is unknown and because recent studies have shown that short exercises or short exposure to a listening task may show improvements similar to long-term training sessions (e.g., Tye-Murray et al., 2017), exercises in the current design were divided into blocks and sessions and were administered in a period of 3 weeks. The exercises included tasks in quiet and noisy listening conditions, and tasks of detection, discrimination, identification and comprehension, taking into consideration the model of Erber and Hirsh (1978). This model presents synthetic and analytic activities that induce bottom up and top down auditory processes (Schow and Nerbonne, 2007). It focuses on a range of auditory skills, including auditory detection, auditory discrimination, auditory identification and comprehension. Exercising on these auditory skills uses syllables, words, phrases, sentences, and connected speech. Our motive from this auditory program model was to reflect performance on different tasks. For example, the listener is asked to determine if two presented words are the same or different. On the other hand, speech comprehension tasks reflect a top down (synthetic) approach, and allows the listener to take advantage of contextual and syntactical cues provided by connected speech (Olson, 2015). Successful auditory program in this population would foster the effective and efficient trading of bottom up and top down processing (Pichora-Fuller and Levitt, 2012). In addition, exercising on several auditory skills with varying level of difficulty could overcome the null effects of the generalization seen in previous protocols, and promote improvements of the trained tasks and transfer of learning (Whitton et al., 2014, 2017; de Larrea-Mancera et al., 2022).

Specifically, we evaluated the combination of auditory exercises and use of HAs on speech in noise perception outcomes and subjective outcomes in the use of HAs through the first month of the adaptation to new HAs. A group of new HA users underwent the auditory program, as well as a group of experienced users, and a group of participants who never used hearing aid use. The suggested exercises protocol includes tasks ranging from syllable identification to comprehension of live speech and assesses speech perception in quiet and in noisy listening conditions in the same study design.

## Materials and Methods

### Participants

A total of 60 Arabic speakers, 40–60 years old, with mild-to-moderate symmetrical sensorineural hearing loss participated in the study. The participants were divided into 3 groups depending on the use of HAs. Group 1: Experienced HA users (referred to as experienced users), who used bilateral HAs 6 h/day (*M* = 9.3 h/day, SD = 2.6), daily for 2–3 years (*M* = 2.3 years, SD = 0.41). Group 2: New HA users (referred to as new users), who were fit for the first time with bilateral HAs and received auditory exercises within 2 weeks after initial fitting. Group 3: Non-HA users, who never used HAs served as the control group for HA use (referred to as non-users). The non-users recruited for this study were individuals who came in for audiological assessments but did not purchase a hearing aid. Specific to this study group, participants reported they were not ready for a HA, cosmetic issues, financial issues, and some reported they did not feel they needed them.

Inclusion and exclusion criteria: All the new and experienced participants used bilateral receivers in the ear canal. All participants had mild-to-moderate pure-tone average (PTA) across frequencies: 500, 1,000, 2,000, and 4,000 Hz (American National Standards Institute, 2010). Adults with neurological or mental disorders or conductive or asymmetrical hearing losses were not included. The participants were recruited from a large clinic in Jerusalem, after signing informed consent to participate. The study was approved by the Ethics Committee of the University of Haifa.

Data from 53 participants were included in the present analysis as seven were excluded. One could not complete all the sessions due to COVID-19 infection; 1 lost his HA during the program period, 3 did not understand the instructions and mistakenly repeated the tasks on their own; therefore, their results could not be used in the analysis. 2 participants were lost to follow-up.

A power analysis using G*Power software (v.3.1.9.2) determined that a sample size of 16 participants would power the study at 80% to detect small-to-medium sized effects (Cohen’s *d* = 0.19, partial η*2* = 0.035) at α = 0.05 for repeated-measures ANOVA. The final numbers in each group were 17 experienced users, 19 new users and 17 non-users.

#### Hearing Aids

Hearing aids were programmed with real ear measurements via the NOAH-LINK platform connected to a desktop computer. The perspective rule NAL-NL2 was used for HA programming and the gain was positioned based on the auto surround program gain (which is automatic gain control and MPO). The data logging feature was always on, in order to check the average duration of use. All participants were fitted bilaterally with Hansaton sound stream SHD (with level technology 3, only one user had level 5 technology) receiver in the canal HAs, with size M receivers and open domes. These were used to facilitate patient comfort and compliance with the HAs. Fitting was conducted in general based on Kuk and Keenan (2006) and Winkler et al. (2016), however, as stated in Winkler et al. (2016), modifications were sometimes needed for individual fitting. Therefore, based on REM, modifications were made to reach the target as much as possible even when using open domes. New users were encouraged to wear their hearing aids for at least 8 h a day. The hearing aid use (average hours/day) was monitored through the hearing aid data logging function (*M* = 5.8 h/day, SD = 1.42). As can be seen the New-users average use of hearing aids was lower than the average use by the experienced users [*t*(34) = 5.03, *p* = 0.001]. Participants were unable to alter the hearing aid gain to minimize variability. Noise reduction programs were turned off during the exercises period for the new and experienced users [similar to the protocol used in Karawani et al. (2018a)].

### Study Design

**FIGURE 1:**
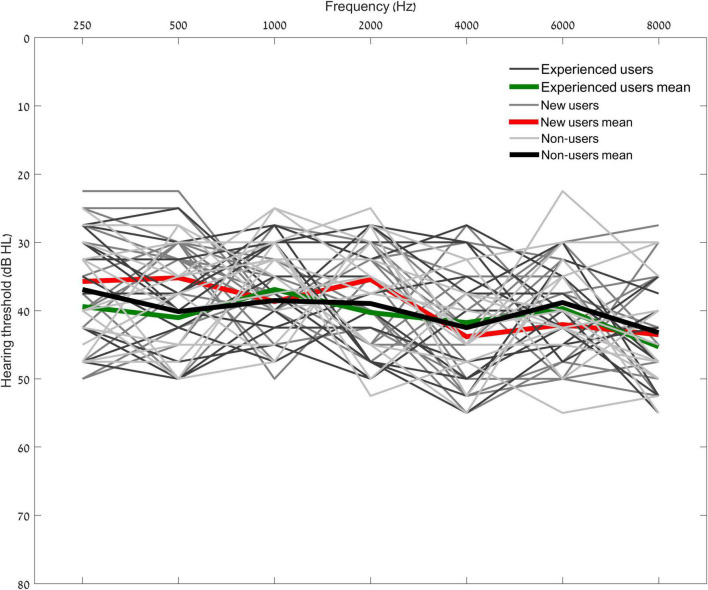
Audiograms. Individual and Mean unaided hearing thresholds (dB HL) (average of both ears) are plotted for the experienced users (green), new users (red), and non-users (black). No significant differences were observed in any of the tested frequency (*p* > 0.07) or the pure tone average (PTA) (Experienced users PTA = 40 dB HL, SD = 5.5; New users PTA = 38 dB HL, SD = 3; Non-users PTA = 39.93 dB HL, SD = 4.6; *p* = 0.31) of each group.

**FIGURE 2:**
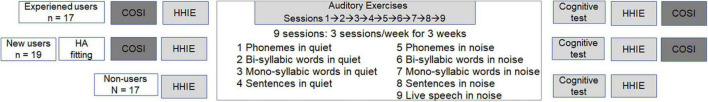
Study design. Hearing aid candidates were fit with bilateral hearing aids and assigned to the new users group (*n* = 19). Experienced users (*n* = 17) and new users filled out the Client Oriented Scale of Improvement (COSI) and the Hearing Handicap Inventory for elderly (HHIE) questionnaires and the non-users (*n* = 17) filled out the HHIE questionnaire. All participants trained at home for ∼30 min per session, at three sessions per week for 3 weeks. Overall they underwent nine sessions, four sessions in quiet and five session under background noise, as listed in the figure. After completion of the exercises program, the participants underwent a cognitive test, and filled again the questionnaires.

After signing the consent form, all potential participants were evaluated in the clinic, to determine whether they meet the inclusion criteria (severity of hearing loss, age, use of HA, type of HA, duration of wearing HA, and other disorders). All participants were in the mild-to-moderate range (Clark, 1981) of SNHL (Figure 1). Each participant was assigned to the appropriate group: experienced users, new users, and non-users. The HA groups completed two questionnaires, and the non-users one questionnaire, detailed in the Self-Assessment Questionnaires below. All participants underwent nine sessions of exercises, three sessions/week for a period of 3 weeks (detailed procedure presented in see section “Procedure”) and a cognitive test at the end. After completing the auditory program, participants were asked to repeat the questionnaires. Figure 2 is a flow chart of the study design.

### Experimental Materials

#### Self-Assessment Questionnaires

The questionnaires were presented and completed in Arabic. All participants completed the Hearing Handicap Inventory for elderly (HHIE) (Ismail et al., 2020), which is a self-assessment tool containing 25 questions assessing the impact of hearing loss on the emotional and social-situational adjustments, before and after the auditory program. Each question had three answers; (1) yes, (2) sometimes or (3) no. Each “yes” answer receives four points, “sometimes” two points and 0 points for “no.” Scores before (pre) and after (post) the auditory program were calculated for the emotional and situational sections.

Only the experienced and new users filled out the Client Oriented Scale of Improvement (COSI) (Dillon et al., 1997; Emerson and Job, 2014) before and after the auditory program. This is a measure of HA benefit that helps patients determine listening situations where improved hearing ability is needed. Five situations were considered for this specific measure: “Conversation with 1 or 2 in Noise,” “Conversation with Group in Noise,” “Hear Front Door Bell or Knock,” “Increased Social Contact,” “Feel Embarrassed.” Possible responses were hardly ever: (which scores 10%), occasionally: (which scores 25%), half of the time: (which scores 50%), most of the time: (which scores 75%), and almost always: (which scores 95%). The percentages for each situation for each participant was calculated and represented the score for between participant comparisons and within participant comparisons before (pre) and after (post) auditory program.

#### Cognitive

The cognitive test was conducted in a quiet listening condition at the end of the last session of the auditory program. The experienced and the new users conducted this test while wearing their HAs. Digit span forward and backward from the Weschler Adult Intelligence Scale-III (Wechsler, 1997) were tested. Each participant was asked to recall an increasing length of digits immediately after auditory presentation. The digits were presented at a rate of one per second. The forward digit span task measures short-term memory capacity, whereas the digit span backward task depends more on attention and working memory skills. In the digit span backward, the participants were asked to repeat numbers in the reverse order of that presented by the examiner. In these subtests, each correct response was worth one point, with a maximum of 16 for the digit span forward task (16 sets), and 14 for the digit span backward subtest (14 sets). In each task, raw scores of the correct answers were added together and calculated. The scores for the forward and backward subtests were used to examine group differences.

#### Perceptual Auditory Exercises Program

##### Stimuli

All stimuli were recorded by a female native Arabic speaker in a soundproof booth with an audible voice using natural speech and intonation. The Jerusalem dialect, which is the dialect of the participants, was used. All recordings were developed using the Mindstamp program (Interactive Video Platform, AECH CO). A pilot was conducted for each recorded session on five normal-hearing adults to verify the trials. The stimuli used were phonemes, monosyllabic words (Abdulhaq, 2006), bi-syllabic words (used in Ratcliff, 2006; Bsharat-Maalouf and Karawani, 2022a,b), sentences, passages of four sentences and connected speech, as detailed in Supplementary Table 1.

##### Protocol

The home-based auditory program took place in the participants’ homes. Internet links were sent to the participants for each session. The program consisted of nine sessions over a course of 3 weeks (as illustrated in Figure 2). There were three sessions (delivered on Monday, Wednesday, and Friday), each week, and each lasting 35–40 min. The last session was conducted via Zoom software (Zoom Video Communications, Inc.). The program started with sessions in quiet listening conditions (sessions 1–4), and then in background noise (5–9) of 4-talker babble noise (two females and two males) at a fixed level of signal-to-noise ratio of 0 dB. The sessions included: (1). Phonemes stimuli in quiet. (2). Bi-syllabic words in quiet. (3). Mono-syllabic words in quiet. (4). Sentences in quiet. (5). Phonemes stimuli in noise. (6). Bi-syllabic words in noise. (7). Mono-syllabic words in noise. (8). Sentences in noise. (9). Live speech with background noise. Each session consisted of four tasks based on the model of Erber and Hirsh (1978). Sessions included the following tasks: detection (participants had to detect the presence of a sound), discrimination (determine whether sounds were the same or different), identification (participants had to identify the signal that was presented), and comprehension tasks (to understand the meaning of the presented word/sentence). The program started with these low difficulty tasks, such as detection, to motivate, captivate, and connect with the participants to achieve commitment. Each task in each session included 30 trials; starting with 30 trials in task 1, then moving to another 30 in task 2, task 3 and finally task 4 (30 × 4 = 120 trials/session). Details of each task and the stimuli presentations are shown in Supplementary Table 1.

##### Procedure

The auditory exercises were done while wearing HAs (for the users groups). Stimuli were presented in sound field via two speakers (either Creative Inspire T 10 2.0 or Logitech Z130 2.0) placed on either side of the computer and facing the participant in a 45°, similar to the protocol in Karawani et al. (2016). Additionally, we ensured that the participants had Zoom installed on their computers and that they understood the Mindstamp program. The participants were asked to adjust the sound to their most comfortable level, where they heard the signal clearly (not too loud and not too soft). Recorded sessions were sent to participants via an internet link. Responses for completed sessions were saved and sent directly to the examiner through the Mindstamp program, and then the examiner scored these responses accordingly. At the beginning of each session and each block, instructions were provided to the participants and examples were provided.

##### Scoring

For each trial listed above, a correct answer received 1 point, and each incorrect answer received 0 points. The scores of each participant in each task and across the four tasks of each session were calculated. The average percentage of correct responses for each session was calculated, referred to as accuracy, and used for the statistical analysis for within participant comparisons (across sessions) and between participant comparisons (across groups).

### Statistical Analyses

Statistical analyses were performed using SPSS software. Between and within participant comparisons were conducted for the self-assessment outcomes, cognitive outcomes and for the exercises outcomes. All statistical analyses were adjusted for multiple comparisons using Bonferroni corrections (Abdi, 2007) and reports are provided after adjustments. Shapiro-Wilk tests were used to confirm that the data were normally distributed (*p* > 0.1).

Self-Assessment HHIE – Between participant comparisons were conducted by comparing subjective responses (self-report emotional score and situational emotional score) across the three study groups (experienced users, new users, non-users) and within participant comparisons were examined by comparing scores before and after the program, using time as the within participant factor (pre-, post-). *Post hoc* tests were also conducted to compare between groups when relevant.

Self-Assessment COSI – Between participant comparisons were conducted by comparing subjective responses (self-report scores for the five listening situations) across the experienced and new HA users’ groups. Within participant comparisons were examined by comparing scores before and after the program, using time as the within participant factor (pre-, post-). *Post hoc* tests were also conducted when relevant.

Cognitive – Between participants comparisons were conducted by comparing the cognitive scores for the forward and backward subtests across the three groups.

Exercises sessions – Between participant comparisons were conducted by comparing perceptual performance (accuracy) across the three groups and within participant comparisons were examined by comparing performance (accuracy) across the different tasks. To examine effects of the listening condition, accuracy in the sessions in quiet were compared to those in noise by adding the within participant factor (condition: quiet, noise). This analysis enabled comparisons for the tasks presented in 1 vs. 5; 2 vs. 6; 3 vs. 7; and 4 vs. 8. Task 9 was analyzed alone. *Post hoc* tests were conducted to compare between groups when relevant.

## Results

### Subjective Outcomes

#### Hearing Handicap Inventory for Elderly (HHIE)

**FIGURE 3:**
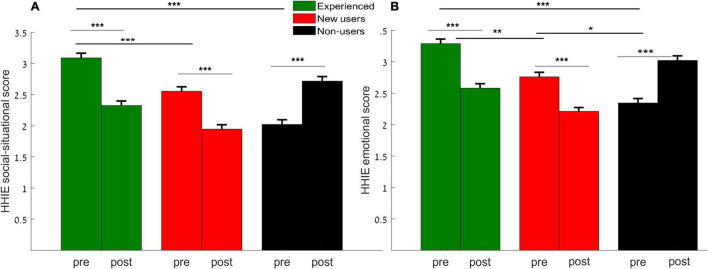
Hearing Handicap Inventory for elderly (HHIE) mean score for the experienced (green), new users (red) and non-users (black) obtained from the pre- and post- reports across the social-situational questions **(A)**, and the emotional questions **(B)**. Error bars represent standard error of the mean. Lines indicate between group comparisons (black lines) and within group (gray lines) comparisons, *p < 0.05, **p < 0.01, ***p < 0.001.

Repeated measures ANOVA for a between participant factor (group: experienced users, new users, non-users) and within participant factor (time: pre-, post-) for the situational and emotional reports revealed a main effect of group [*F*(2,50) = 28.319, *p* < 0.001, η^2^*p* = 0.531] (where the experienced users showed the higher scores), a main effect of time [*F*(2,49) = 10.132, *p* < 0.001, η^2^*p* = 0.293], where lower scores were reported post-program, and a significant time × group interaction [*F*(2,50) = 49.729, *p* < 0.001, η^2^*p* = 0.791] was observed. *Post hoc* tests with Bonferroni corrections revealed significant differences in the overall scores between groups (experienced vs. new users, *p* < 0.01, experienced vs. non-users, *p* < 0.001), as can be seen in Figure 3 for the *situational* (A) and for the *emotional* scores (B), with overall higher scores obtained in the experienced users’ group. In addition, significant difference was observed between the new and non-users in the emotional score (*p* = 0.032) and a marginal significant effect in the situational score (*p* = 0.052), probably due to the opposite effect seen in non-users, discussed below. The experienced users and the new users showed better scores in their self-reports in the post-test. While the non-users reported worse scores in the post-test, this will be discussed below.

#### Client Oriented Scale of Improvement (COSI)

**FIGURE 4:**
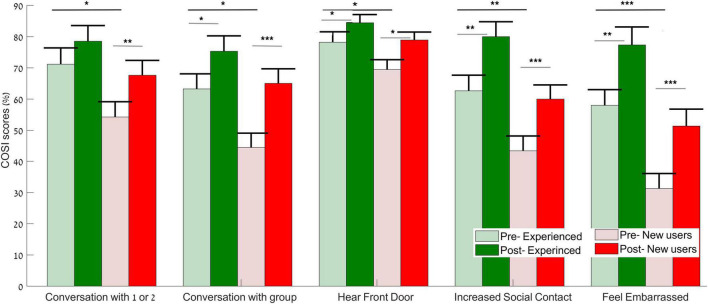
Client Oriented Scale of Improvement (COSI) mean score (%) for the experienced (green) and new users (red) obtained from the pre- (light bars) and post- (dark bars) reports across the five listening situations: “Conversation with 1 or 2 in Noise,” “Conversation with Group in Noise,” “Hear Front Door Bell or Knock,” “Increased Social Contact,” “Feel Embarrassed.” Error bars represent standard error of the mean. Dark lines indicate main group effects as indicated by ANOVA- overall scores. Gray lines indicate within group comparisons, *p < 0.05, **p < 0.01, ***p < 0.001.

Repeated measures ANOVA for between participant factor (group: experienced users, new users) and within participant factor (time: pre, post) across the five COSI situations revealed a main effect of group [*F*(5,30) = 5.437, *p* = 0.001, η^2^*p* = 0.475], with higher scores for the experienced users than the new users. There was a main effect of time [*F*(5,30) = 25.842, *p* < 0.001, η^2^*p* = 0.812], with higher scores in the post- compared to the pre reports (Figure 4). A significant time × group interaction (*p* = 0.636) was not observed. This non-significant interaction indicates that the pre – post changes were similar between the groups (Figure 4). Univariate ANOVAs for each situation followed by *t*-test analysis to reveal pre – post changes in COSI situations in the experienced and in the new users’ groups are shown in Supplementary Table 2.

### Cognitive Test Results

**FIGURE 5:**
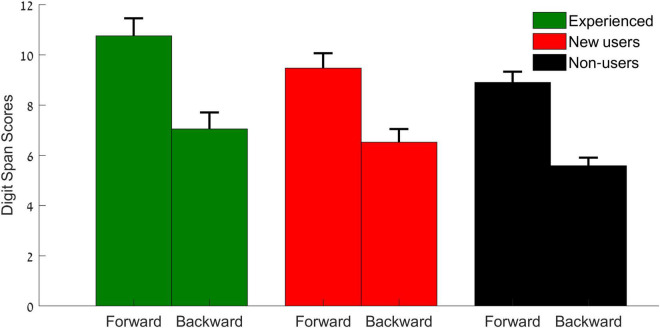
Digit span forward and backward scores for the experienced users (green), new users (red), and non-users (black). Error bars represent standard error of the mean.

Repeated measures ANOVA for between participant factors (experienced users, new users, non-users) and within participant factors (subtest: forward, backward) revealed a subtest main effect [*F*(1,50) = 190.465, *p* < 0.001, η^2^*p* = 0.792], where scores on the forward tests were higher than the backward subtest (Figure 5). A main effect of group was observed [*F*(2,50) = 4.111, *p* = 0.049]. No subtest × group interactions (*p* = 0.091) were observed.

### Task Accuracy

**FIGURE 6:**
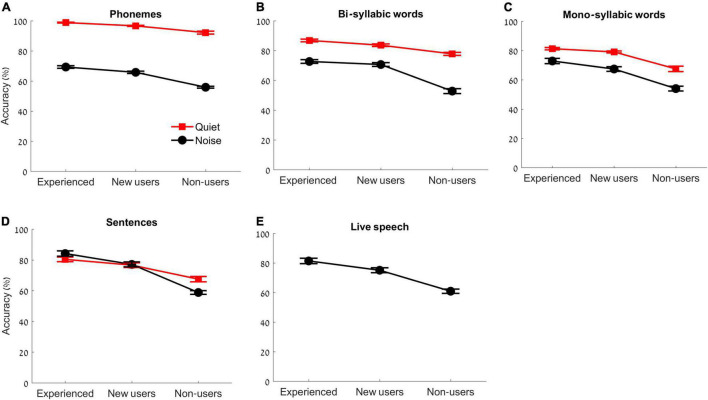
Task accuracy (%) for the experienced users, new users, and non-users, across the following tasks: **(A)** Phonemes, **(B)** Bi-syllabic words, **(C)** Mono-syllabic words, **(D)** Sentences in both quiet (red) and in noise (black) and for **(E)** Live speech. Error bars represent standard errors of the mean.

Repeated measures ANOVA for a between participant factor (group: experienced users, new users, non-users) and two within participant factors (condition: quiet, noise; and task: 4 levels) revealed a main effect of task [*F*(3,50) = 66.732, *p* < 0.001, η^2^*p* = 0.572], indicating that participants had higher performance on simpler tasks, a main effect of condition [*F*(1,50) = 674.391, *p* < 0.001, η^2^*p* = 0.931] indicating higher performance in quiet than in noise conditions, a main effect of group [*F*(2,50) = 216.338, *p* < 0.001, η^2^*p* = 0.896], indicating the highest performance was observed in experienced users and lowest accuracy in the non-users (Figure 6). In addition, a task × condition interaction was observed [*F*(3,50) = 168.312, *p* < 0.001, η^2^*p* = 0.771] indicating overall higher accuracy in earlier (simpler) tasks and in quiet. A task × group interaction was observed [*F*(6,50) = 5.859, *p* < 0.001, η^2^*p* = 0.190], where the non-users obtained the lowest scores, especially in later (more complex) tasks. A condition × group interaction was observed [*F*(2,50) = 19.751, *p* < 0.001, η^2^*p* = 0.441], where the non-users had the lowest scores – especially in noise. A condition × task × group interaction was not significant [*F*(6,50) = 2.028, *p* = 0.06]. This indicates that the non-users performed significantly worse in noisy conditions and there was no specific task in which the group performed differently. Results are illustrated in Figure 6.

To understand the group effects in each of the exercises (1–9), univariate ANOVAs were conducted followed by *post hoc* pairwise comparisons (using *t* tests). The statistics for these measures are reported in Table 1 for convenience, as well as the trend of the effect (which group showed the higher accuracy). Adjustments for multiple comparisons were made using Bonferroni corrections (as reported in the see section “Materials and Methods”). In general, the non-users had the lowest accuracy across all tasks (as can be seen in Table 1). The new users performed better than the non-users in all tasks (*p* < 0.001), whereas in some tasks the new users’ performance was similar to that of the experienced users (as seen in Table 1, in tasks 1, 2, 3, 4, 6, and 7, *p* > 0.051), and in other tasks their performance was lower than that of the experienced users (in 5, 8, and 9, *p* < 0.029).

**TABLE 1:** Task accuracy.

		Univariate ANOVA	Pairwise comparisons			
		Main effect of group	Experienced vs. New users	Experienced vs. Non-users	New vs. Non-users	Description of the trend of effect
1	Phonemes in quiet	*F* = 31.928 *P* < 0.001	*P* = 0.05 [0.10, 4.01]	*P* < 0.001 [4.56, 8.78]	*P* < 0.001 [2.45, 6.56]	Experienced = New > Non-users
2	Bi-syllabic words in quiet	*F* = 23.847 *P* < 0.001	*P* = 0.052 [−0.024, 6.37]	*P* < 0.001 [5.73, 12.31]	*P* < 0.001 [2.64, 9.04]	Experienced = New > Non- users
3	Mono-syllabic words in quiet	*F* = 37.630 *P* < 0.001	*P* = 0.557 [−1.899, 6.388]	*P* < 0.001 [9.56, 18.08]	*P* < 0.001 [7.43, 15.73]	Experienced = New > Non-users
4	Sentences in quiet	*F* = 16.976 *P* < 0.001	*P* = 0.287 [−1.72, 9.26]	*P* < 0.001 [7.24, 18.54]	*P* < 0.001 [3.62, 14.62]	Experienced = New > Non-users
5	Phonemes in noise	*F* = 81.479 *P* < 0.001	*P* = 0.005 [0.88, 6.16]	*P* < 0.001 [10.72, 16.14]	*P* < 0.001 [7.27, 12.54]	Experienced > New > Non-users
6	Bi-syllabic words in noise	*F* = 61.181 *P* < 0.001	*P* = 0.895 [−2.76, 6.84]	*P* < 0.001 [14.96, 24.83]	*P* < 0.001 [13.05, 2.66]	Experienced = New > Non- users
7	Mono-syllabic words in noise	*F* = 32.521 *P* < 0.001	*P* = 0.071 [−0.33, 11.29]	*P* < 0.001 [12.89, 24.84]	*P* < 0.001 [7.57, 19.21]	Experienced = New > Non-users
8	Sentences in noise	*F* = 69.863 *P* < 0.001	*P* = 0.006 [1.71, 12.41]	*P* < 0.001 [19.84, 30.83]	*P* < 0.001 [12.93,23.63]	Experienced > New > Non-users
9	Live speech	*F* = 38.472 *P* < 0.001	*P* = 0.029 [0.51, 12.07]	*P* < 0.001 [14.55, 26.42]	*P* < 0.001 [8.42, 19.98]	Experienced > New > Non-users

*Summary of the statistical differences across groups and tasks indicated by Univariate ANOVAs. Main effect of group is indicated by F(2,52) statistic values for each task. Pairwise comparisons for the group effects were reflected by P values and [95% confidence intervals]. Description of the trend of effect is also provided. “ > ” indicates higher accuracy; “ = ” indicates non-significant difference.*

## Discussion

The current study examined perceptual auditory speech performance of middle-aged adults with a range of mild-to-moderate sensorineural hearing loss, who were experienced users, new users, or non-users of HAs through a home-based perceptual auditory exercises program. The study examined self-reported hearing ability after this period of the auditory program. The program focused on examining the perceptual differences in the ability to recognize and discriminate speech in both quiet and noisy conditions across groups. Differences in performance across groups in quiet and noisy conditions were observed. The non-users had lower accuracy across all speech perception sessions compared to the users’ groups. The performance of all groups deteriorated in noise. However, the experienced users had the least deterioration. The subjective measurements revealed that new HA users reported subjective improvements within a month of using HAs; although overall, subjective reports were higher in the experienced users.

### Self-Assessment Reports Across Groups

Changes in self-reported hearing ability after the auditory exercises were reflected in subjective reports using HHIE and COSI questionnaires. In both the situational and the emotional sections, the HHIE showed improvements between the pre- and post-exercises reports in the users groups. Subjectively, the experienced users reported higher scores than new users and higher than the non-users. Our results are consistent with previous studies that utilized the HHIE questionnaire and found improved skills post-auditory-exercises (Magalhães and Iório, 2011; Guarinello et al., 2013; Teixeira and Costa-Ferreira, 2018). However, additional studies with a control group of untrained HA users and non-users is needed to verify the current findings. Fostick et al. (2020) also showed that after auditory training, older adults (even non-rehabilitated adults; in our case non-users) demonstrated improvements in self-efficacy. The current study’s finding showed an opposite effect in the non-users group; where they reported worse scores after the exercises period. This might be explained by the fact that they acknowledged their deficit in a more positive way at the beginning, maybe because they were sure that they do not need a hearing aid. Perhaps after the period of the exercises, especially in noise, they might have felt the difficulty, and not to mention being in lockdown due to COVID situation, so perhaps these led them to self-report their “real” ability in the post-test, and thus show that they have lower subjective reports.

As indicated by the COSI, both HA groups evaluated themselves with higher scores after the exercises in comparison to the pre-exercises scores, in situations that reflect everyday communication difficulties. These results are consistent with those of Tye-Murray et al. (2016), where participants rated themselves as having improved communication in their daily life after training. New users demonstrated subjective improvement after using HAs for 1 month. However, as can be seen from the results in social and embarrassed situations, overall, the reports of the new users were not as high reports as those of the experienced users (Figure 4). A study by Shabana et al. (2017) indicated that HAs improved social contact; however, users still felt embarrassed and were rated in COSI by experienced users as a high priority. Because expectations of first-time HA users are often unrealistic (Wong et al., 2003; Meyer et al., 2014), we suggest that interventions are needed, other than speech in noise training, to overcome social difficulty. Recent studies have suggested that an educational intervention before HA-fitting could yield better acceptance of the HAs (Maidment et al., 2020; Ferguson et al., 2021).

Our results are consistent with studies of Karawani et al. (2018a), and Nkyekyer et al. (2019) in which decreased self-reported disability was observed when using hearing aids. Earlier studies by Olson et al. (2013) and Dawes et al. (2014) indicated that self-reported scores revealed that only the new users improved and not the experienced users. Methodological differences might be the reason for the different results between studies. In addition, a control group of HA users with no exercises program is needed to test possible effects delivered by expectations of the participants being biased to the program exercises on the improved subjective ability.

### Speech Perception Across Sessions and Groups

In all sessions, performance differed significantly between the non-users compared to the experienced and new users, suggesting that HAs are needed to perform better in speech perception tasks. However, the picture was not as clear for new HA users compared to experienced users, as differences were not observed between the new and experienced users in all sessions. In quiet listening conditions, performance was similar between the two groups, indicating the immediate improvement from using amplification in quiet situations. Performance differences were reflected more in noisy sessions (specifically in sentences in noise and live speech in noise sessions, Table 1 and Figure 6), demonstrating that a longer period of using HAs may be needed to obtain perceptual benefits during speech perception in noise. Especially that we noted a difference in the daily use of hearing aids between the experienced and the new users groups. These effects should be studied with the addition of objective measures. Further, as can be seen from the non-significant effect in noise, between experienced and new users, in sessions 6 and 7 (Table 1), it seems that the auditory exercises enabled improvement in speech perception tasks. This may be explained by rapid learning through exposure to auditory tasks (Karawani et al., 2017; Banai et al., 2021) and/or because the participants became accustomed to the auditory program procedure using the computer (Tuz et al., 2021). In session 5 (first noise session), differences were again observed between experienced and new users. In session 6, new users showed similar performance in noise as the experienced users did. This is similar to Rao et al. (2017) who reported improvement in speech perception in noise and improvement in auditory selective attention with training, in new users. Because the current study did not assess learning and because our exercises were different, we related the effects seen in the new users to rapid learning due to exposure to auditory tasks, and to the gain in audibility by the amplification provided by the HAs.

Studies have reported that although HAs improve the hearing of people with hearing loss through different technologies (Wu et al., 2019), they do not sufficiently compensate for speech perception abilities despite considerable advancements in digital technologies (McCormack and Fortnum, 2013; Bisgaard and Ruf, 2017; Hoppe and Hesse, 2017; Johnson et al., 2018). Although in the current study, there was no group of new HA users who did not undergo the program, previous reports have shown that speech in noise performance after using HAs for 6 months did not show large perceptual improvements (Karawani et al., 2018a). Even though it is evident that enhanced technology can partially address specific hearing difficulties, additional auditory exercises could be another means of improving speech-in-noise recognition (Anderson and Kraus, 2013; Lessa et al., 2013; Kuchinsky et al., 2014; Yu et al., 2017; Sattari et al., 2020). A recent study by Kang et al. (2020) also showed that after an 8-week auditory training program, with 10 HA users who wore their HAs for more than 10 months, improvements were observed in speech recognition in noisy situations, and in subjective measurements of HA satisfaction. This shows that auditory exercises can be also administered to experienced users. The current study did not assess pre – post objective outcome measures for speech in noise perceptual improvements. The findings suggest that longer experience with using HAs may enable better speech in noise perception, as reflected by group differences.

To summarize, the current study suggests benefits of the use of HAs, as reflected in differences in self-reports and in differences in speech perception scores between users and non-users of HAs, and that overall, the experienced users had higher scores than other participants did. Overall the experienced users had higher scores than the non-users, which supports the findings of Hyams et al. (2018) showing that non-users have lower self-reported abilities than the HA users do. Furthermore, although our study included only one cognitive test, it is known to evaluate working memory. Previous studies connected the difficulty in understanding speech in noise to limited cognitive abilities and especially working memory (Rönnberg et al., 2013; Gordon-Salant and Cole, 2016; Rönnberg et al., 2021). In the current study, the non-users had the lowest scores compared to the experienced users who had higher scores. We should note (as reflected in the methods) that the users’ groups underwent the cognitive test in an aided condition (while using their HAs), while the non-users were in the unaided position, this could have contributed for the overall difference across groups. Delivering a digit span test in a visual modality could have addressed this concern. Therefore, examination of a battery of cognitive measures should be further assessed.

## Conclusion

The current study proposes a home-based auditory exercises program that promotes speech in noise perception by implementing different tasks in noise. This can supplement the diagnostic evaluation, especially when it is not completed because of time limitations of audiologists. The current protocol includes exercises in different tasks, from phoneme perception, to sentences and live speech perception abilities; a study to examine learning benefits of a longer training period with each protocol should be considered. The development of these home-based auditory exercises have considerable potential to expand the type of interventions available and deliver them to older adults in their homes.

## Strengths and Limitations of the Study

The study was conducted in the participants’ homes; they did not need to come to the clinic. In a situation like COVID-19, we also worked on their auditory abilities without risking their health or that of the staff. The study analyzed speech perception across sessions, and not the progression of pre- and post-learning effects. The sample size was relatively small, and time limitations were an issue. The current study did not include cognitive training tasks (only one cognitive test was used). It is important to monitor cognitive abilities with the use of auditory training protocols (Stropahl et al., 2020). Furthermore, the lack of a group of new and experienced HA users who did not undergo the auditory exercises program is a limitation of the current study. Therefore, such a group will add to the findings. In addition, because of COVID-19 quarantine at the time of data collection, objective post-tests were not collected (which were supposed to take place in the clinic). Therefore, analysis of pre – post objective measures was limited to subjective questionnaires and within program-task analyses. In addition, since participants were in lockdown they might have not experienced noisy conditions outside the house such as in restaurants, etc., and these might have affected the self-reports. Future studies should be conducted that avoid these weaknesses.

## Data Availability Statement

The original contributions presented in the study are included in the article/Supplementary Material, further inquiries can be directed to the corresponding author.

## Ethics Statement

The studies involving human participants were reviewed and approved by the Ethics Committee of the Faculty of Social Welfare and Health Sciences, University of Haifa. The patients/participants provided their written informed consent to participate in this study.

## Author Contributions

HKarah and HKaraw designed the study, analyzed the data, interpreted the results, and wrote the manuscript. HKarah collected the data. HKaraw provided funding, supervision, and review. Both authors contributed to the article and approved the submitted version.

## Conflict of Interest

The authors declare that the research was conducted in the absence of any commercial or financial relationships that could be construed as a potential conflict of interest.

## Publisher’s Note

All claims expressed in this article are solely those of the authors and do not necessarily represent those of their affiliated organizations, or those of the publisher, the editors and the reviewers. Any product that may be evaluated in this article, or claim that may be made by its manufacturer, is not guaranteed or endorsed by the publisher.
