# Supplementary material for: Auditory Perceptual Exercises in Adults Adapting to the Use of Hearing Aids
Source: Front Psychol. 2022 May 18;13:832100. doi: 10.3389/fpsyg.2022.832100 (PMC9158114; doi:10.3389/fpsyg.2022.832100)
Supplement: Supplementary file 1 [file Data_Sheet_1.PDF]

Supplementary Material

Supplementary Table S1. Auditory training sessions and task details. Sessions 1-3 were conducted in the first week, sessions 4-6 in the second week, and sessions 7-9 in the third week. Listening sessions with background noise are highlighted in grey.

**Session #**	**Session title**	**Task 1**	**Task 2**	**Task 3**	**Task 4**
**1**	**Phonemes in quiet**	In each trial, you will hear pairs of *sounds* (*phonemes*). Following each pair, one additional sound will be presented. If you hear the same sound that was presented in one of the pairs, you have to click the answer yes, if you did not hear the sound, then click the answer "no".	In each trial, you will hear a pair of *sounds*, and you have to discriminate if they are the same or different. If same, you have to click the "yes" answer, if different then click the "no" answer.	In each trial, you will hear a *sound*, and then you need to choose the sound that was heard from the list.	In each trial, you will hear a *sound that represents a letter* (phoneme), and then you need to write the letter in the response box
**2**	**Bi-syllabic words in quiet**	In each trial, you will hear pairs of *words*. Following each pair one additional word will be presented. If you hear one of the words that were previously presented, you have to click the answer yes, if you did not hear the sound, then click the answer "no".	In each trial, you will hear a pair of *words*, and you have to discriminate if they are the *same or different.* If same, you have to click the "yes" answer, if different then click the "no" answer.	In each trial, you will hear a *word*, and then you need to choose the word that was heard from the list.	In each trial, you will hear a *word* and then you need to type the word in the response box.
**3**	**Mono-syllabic words in quiet**	In each trial, you will hear pairs of *words*. Following each pair one additional word will be presented. If you hear one of the words that were previously presented, you have to click the answer yes, if you did not hear	In each trial, you will hear a pair of *words*, and you have to discriminate if they are the *same or different.* If same, you have to click the "yes" answer, if different then click the "no" answer.	In each trial, you will hear a *word*, and then you need to choose the word that was heard from the list.	In each trial, you will hear a *word* and then you need to write the word in the response box

		the sound, then click the answer “no”.			
**4**	**Sentences in quiet**	Answer the questions please. Type the response in the box	Sentence identification (closed set)<br>In each trial, a sentence will be presented, and then you need to choose the sentence that was heard from the list.	In each trial, a short story will be heard. After each story, you will need to sequence the events correctly in the right order.	In each trial, you will hear a short passage.<br>You will need to answer the question relevant to the passage.<br>[Type in your response]
**5**	**Phonemes in noise**	In each trial, you will hear pairs of *sounds* (*phonemes*) presented in background noise. Following each pair, one additional sound will be presented. If you hear the same sound that was presented in one of the pairs, you have to click the answer yes, if you did not hear the sound, then click the answer “no”.	In each trial, you will hear a pair of *sounds*, presented in background noise and you have to discriminate if they are the same or different.<br>If same, you have to click the “yes” answer, if different then click the “no” answer.	In each trial, you will hear a *sound, presented* in background noise and then you need to choose the sound that was heard from the list.	In each trial, you will hear a *sound that represents a letter* (phoneme), this will be presented under background noise and then you need to write the letter in the response box
**6**	**Bi-syllabic words in noise**	In each trial, you will hear pairs of *words presented in background noise*. Following each pair one additional word will be presented. If you hear one of the words that were previously presented, you have to click the answer yes, if you did not hear the sound, then click the answer “no”.	In each trial, you will hear a pair of *words, presented in background noise*. You need to discriminate if they are the *same or different.*<br>If same, you need to click the “yes” answer, if different then click the “no” answer.	In each trial, you will hear a *word, presented in background noise*. You need to choose the word that was heard from the list.	In each trial, you will hear a *word presented in background noise* and then you need to write the word in the response box
**7**	**Mono-syllabic**	In each trial, you will hear pairs of *words presented in background noise*. Following	In each trial, you will hear a pair of *words, presented under background noise* and you have	In each trial, you will hear a *word presented in background noise*, and then	In each trial, you will hear a *word presented in*

	**words in noise**	each pair one additional word will be presented. If you hear one of the words that were previously presented, you need to click the answer “yes”, if you did not hear the sound, then click the answer “no”.	to discriminate if they are the *same or different.*<br>If same, you have to click the “yes” answer, if different then click the “no” answer.	you need to choose the word that was heard from the list.	*background noise* and then you need to write the word in the response box
**8**	**Sentences in noise**	You will hear a question *in background noise.* Answer the questions please.<br>[Type the response in the box]	In each trial, a sentence will be presented *in background noise*, and then you need to choose the sentence that was heard from the list.	In each trial, a short story will be heard, *presented in background noise*. After each story, you will need to sequence the events correctly in the right order.<br>[type in the response]	In each trial, you will hear a short passage *presented in background noise*. You will need to answer different questions related to the passage [type your answer in the response]
**9**	**Live speech**	In each trial, you will hear pairs of *words*. Following each pair one additional word will be presented. If you hear one of the words that were previously presented, you need to click the answer yes, if you did not hear the sound, then click the answer “no”.	Each time, you will hear four words; one of them is odd, and you have to say the odd word out loud (*with background noise*).	Please repeat each sentence after me	Answering questions with background noise.
					Spontaneous conversational speech with background noise.
