# Supplementary material for: Auditory Perceptual Exercises in Adults Adapting to the Use of Hearing Aids
Source: Front Psychol. 2022 May 18;13:832100. doi: 10.3389/fpsyg.2022.832100 (PMC9158114; doi:10.3389/fpsyg.2022.832100)
Supplement: Supplementary file 2 [file Data_Sheet_2.PDF]

Supplementary Material

Supplementary Table S2. Client Oriented Scale of Improvement (COSI) mean score (top) and (standard deviations) (bottom) for the experienced and new users obtained from the pre- and post- reports across the five situations. Between group comparisons are indicated (by p-value) across the 5 situations and testing sessions (pre- and post-training), as well as within group analysis.

		**Conversation with 1 or 2 in Noise**	**Conversation with Group in Noise**	**Hear Front Door Bell or Knock**	**Increased Social Contact**	**Feel Embarrassed**
**Pre-**	**Experienced users**	71.1<br>(18.25)	63.2<br>(15.60)	78.2<br>(11.31)	62.6<br>(22.65)	57.9<br>(25)
	**New users**	54.2<br>(23.76)	44.4<br>(22.97)	69.4<br>(15.27)	43.4<br>(18.34)	31.3<br>(16.15)
	**p- value** (Experienced vs. new users)	0.023	0.008	0.061	0.008	0.001
**Post-**	**Experienced users**	78.5<br>(20.52)	75.2<br>(14.73)	84.4<br>(10.29)	80.0<br>(19.20)	77.35<br>(20.09)
	**New users**	67.6<br>(20.64)	65.0<br>(24.21)	78.9<br>(11.38)	60.0<br>(19.93)	51.3<br>(26.34)

	**p- value** (Experienced vs. new users)	0.122	0.138	0.142	0.004	0.002
**Difference post-pre (p-value)**	Experienced users	0.072	0.016	0.021	0.004	0.003
**Difference post-pre (p-value)**	New users	0.005	0.001	0.019	0.001	0.001
**Difference post-pre (p-value**)	Experienced vs. New users	0.319	0.979	0.084	0.533	0.625
